# Ensuring equity in mental health and psychosocial support during the COVID-19 pandemic and beyond

**DOI:** 10.1186/s13031-023-00500-5

**Published:** 2023-02-19

**Authors:** Andrea Armijos, Annie G. Bonz, Felicity L. Brown, Danielle Charlet, Flora Cohen, M. Claire Greene, Sabrina Hermosilla, Leah E. James, Karine Le Roch, MHPSS Implementation Science Learning Collaborative

**Affiliations:** 1 HIAS, Ecuador, Mariscal Foch, 170143 Quito, Ecuador; 2 HIAS, 1300 Spring Street, Suite 500, Silver Spring, MD 20910 USA; 3 Research and Development Department, War Child Holland, Helmholtzstraat 61-G, 1098 LE Amsterdam, The Netherlands; 4 University Research Co., LLC, 5404 Wisconsin Ave Suite 800, Chevy Chase, MD 20815 USA; 5 Washington University in St. Louis, Brookings Dr, St. Louis, MO 63130 USA; 6 Columbia University Mailman School of Public Health, 722 W 168th St, New York, NY 10032 USA; 7 University of Michigan, 400 4th St, Ann Arbor, MI 48103 USA; 8 Heartland Alliance International, 208 S. LaSalle Street, Suite 1300, Chicago, IL 60604 USA; 9 Action contre la Faim, 14 Boulevard de Douaumont, 75017 Paris, France

**Keywords:** Mental health, Psychosocial support, COVID-19, Adaptations, Equity, Disparities, Humanitarian, Low- and middle-income countries

## Abstract

Populations affected by armed conflict and other humanitarian crises are at elevated risk for mental health problems. While the COVID-19 pandemic has had broadly deleterious effects on livelihoods, economic well-being, and population health worldwide, vulnerable groups have been disproportionately impacted by the pandemic. Providing mental health and psychosocial support (MHPSS) services during these times to vulnerable groups, especially in low- and middle-income countries and humanitarian settings, is essential. In an effort to comply with the public health response to the pandemic and mitigate COVID-19 transmission, significant implementation adaptations were made to service delivery during the pandemic. This short report describes several strategies to ensure that equity was central to these adaptations and public health responses, and provides recommendations for ensuring continuity of this progress post-pandemic. Examples and key lessons learned are given related to strategies to increase access to MHPSS services, improve meaningful stakeholder engagement, develop and support community networks, and implement community-based psychosocial support groups. They come from diverse settings of Bangladesh, Colombia, Ecuador, and Lebanon. The COVID-19 pandemic has highlighted the importance of preventing and treating MHPSS issues. It also has created opportunities for innovative programming to address overlooked problems, improve the quality of services provided, and increase focus on equity. It is vital that we use the momentum and attention generated around MHPSS services during the COVID-19 pandemic to continue to build and improve existing MHPSS services in more equitable ways for vulnerable populations.

## Introduction

Populations affected by armed conflict and other humanitarian crises are at elevated risk for mental health problems [1]. While the COVID-19 pandemic has had broadly deleterious effects on livelihoods, economic well-being, and population health worldwide [2], vulnerable groups have been disproportionately impacted [3]. The disproportionate health and economic impacts of COVID-19 and the associated responses (e.g., lockdowns, physical distancing) interact with pre-existing vulnerabilities to exacerbate health disparities [4, 5], including access to mental health and psychosocial services which are already scarce in humanitarian contexts [6, 7]. Individuals affected by armed conflict and humanitarian crises therefore experience a triple vulnerability to poor mental health: (i) experiencing a humanitarian crisis; (ii) experiencing the worst impacts of the COVID-19 pandemic; and (iii) increased difficulty accessing relevant and responsive health and mental health services [8, 9]

One positive facet of the COVID-19 pandemic is that the importance of mental health and wellbeing needs have been increasingly recognized [10]. As others have noted, it is essential that when seeking to address COVID-19’s mental health impacts, disparities in mental health are also recognized and equity and inclusion are given top priority [11]. Otherwise, the adaptations that are made to ensure continued and greater access to mental health and psychosocial support (MHPSS) services could actually exacerbate rather than alleviate health disparities. Great opportunities exist at present to use this momentum to strengthen mental health systems globally and “Build Back Better” [2, 12], but it is imperative that we also strive to “Build Back Fairer” [13]. This paper will outline experiences of partners in an MHPSS Implementation Science Learning Collaborative conducting implementation research and providing MHPSS services during COVID-19, and offer recommendations to achieve these aims and maintain progress post-pandemic.

The MHPSS Implementation Science Learning Collaborative, started in July 2020 and was convened by University Research Co. LLC and City University of New York Graduate School of Public Health and Health Policy. It consists of more than a dozen research and implementation institutions and two donors (USAID and GIZ), in six countries across four regions. The Learning Collaborative focuses on facilitating collaboration and cross-site learning amongst partners and creates more effective dissemination and advocacy for the uptake of evidence by strategically leveraging members’ networks.

## MHPSS program and service adaptations during COVID-19

In an effort to comply with the public health response to the pandemic and mitigate COVID-19 transmission, significant adaptations were made to MHPSS service delivery in low- and middle-income countries and humanitarian settings. In-person and place-based MHPSS services were downscaled. Many safe spaces for women and children, including schools and emergency shelters, were closed, and many group programs were suspended or moved to alternative platforms [2, 14]. Where programs continued, they had to be adapted to ensure the physical health and safety of participants [14, 15]. Examples of these adaptations include the use of radio programs to disseminate messages about mental health; audio recordings, messages, and chatbots on phones to deliver bibliotherapy and information about COVID-19; establishing helplines for mental health emergencies and interagency referrals; and remote delivery of individual and group psychosocial support, specialized psychotherapy, and medication management [2, 16–18]. Adaptations were also made to maintain and enhance MHPSS capacity building [19, 20]. Increased access to online training allowed additional volunteers and community workers to be trained in Psychological First Aid (PFA) around the world [21, 22]. Remote supervision also proved an effective strategy for supporting providers in the delivery of PFA and scalable psychological and psychosocial interventions (e.g., Problem Management Plus (PM+), interpersonal psychotherapy, etc.) [2, 23, 24].

These adaptations present opportunities for, and challenges to ensuring equity in MHPSS. According to the Health Equity Implementation Framework [45], which was designed to incorporate determinants of health equity into implementation research, any clinical encounter comprises an interaction between patient-level factors, provider-level factors, and characteristics of an innovation. This in turn is influenced by health-system and societal factors, to determine the success of implementation, and improvements in health equity. Below we explore themes related to this framework through a series of case examples that describe adaptations made by local and international non-governmental organizations and academic institutions to engage stakeholders, ensure access, increase reach, and maintain MHPSS services.

## Equity in consultation and co-design processes for MHPSS services: inclusion and meaningful participation of hard-to-reach stakeholders using technology

Remote activities, necessitated by the COVID-19 pandemic, presented excellent opportunities for increased inclusion and participation of colleagues and stakeholders across the globe in the consultation and design of MHPSS programs. Project and team meetings that were previously attended in person with only team members from a particular location often shifted to an online modality, and were made accessible to others in various locations. If this opportunity is appropriately harnessed and sustained, even after face-to-face activities resume, it has the potential to enhance cross-site learning, move towards equitable access to knowledge and dialogue, and promote deeper contextual understanding of work across settings [25, 26]. All of these can enhance innovation and system level relevance, quality, and equity in participation and decision-making for MHPSS research and programming. As increased access to vaccines allows for individuals to gather in person, this advantage may be lost either due to a failure to prioritize continued inclusion of people from other locations or ineffective hybrid meeting strategies that create two classes of participation. Care must also be taken to ensure costs and burdens associated with inclusion—for example, connectivity costs and convenience of meeting schedules—are appropriately and fairly allocated.

As an example of increasing participation through online activities, UNICEF and WHO involved young people from over 15 countries and multiple regions in joint online sessions to support the co-development of COVID-19 related MHPSS messaging. Through a series of consultations, young people from around the world worked together to identify how best to design and present key messages. Staff from organizations working with hard-to-reach young people helped identify who could participate, and tried to ensure they had access to the necessary digital technology to connect to sessions, for example by supporting them to set up and learn to use video conferencing software [27].

While technology can expand access and facilitate cross-country engagement, it can also present significant barriers to participation compared to face-to-face consultation for those who may not have access due to limited digital connectivity, language barriers, and technological literacy. An approach to engage stakeholders with limited connectivity in the design of services was piloted by War Child Holland and American University of Beirut in Lebanon. The project team gathered input from a community advisory board (CAB) made up of families from low-resource communities in Lebanon to inform the development of a new family-focused MHPSS intervention. Initially the team met with families in person to explain the project and gather early input for the design of the intervention. Soon after, in-person meetings were prohibited due to COVID-19 and securing data and appropriate devices for these families to join online group meetings proved challenging and costly, especially during periods of full lockdown. However, families indicated high levels of comfort with voice notes (on WhatsApp) to communicate, including about sensitive topics. The team sent CAB members questions about the intervention materials and study design via voice note (to overcome literacy issues), they were able to listen at a time that suited them and reply at their convenience. Garnering input from adolescents remained a challenge as respondents and owners of mobile devices were typically parents. It is important to develop short- and long-term solutions to ensure that the voices of the whole family, including those holding less access to resources, can be included confidentially and safely.

These examples provide guidance as to feasible and generally acceptable methods to enable service user participation during crisis contexts such as the COVID-19 pandemic; however, further research is needed to determine optimal platforms for high quality, long term sustainment of inclusive consultations that ensure adequate access, while reducing the burden and ensuring trust, privacy, and confidentiality.

## Equity in access to MHPSS services: leveraging community networks and flexible implementation in Ecuador

Although remote-delivered MHPSS and related activities have the potential to increase access—as was seen in the examples above—there are also populations who may experience reduced access due to limited technological literacy, capacity, or availability of internet and equipment. These populations may also be those for whom MHPSS access is already most limited. To avoid exacerbating potential inequities, flexible implementation of remote-delivered strategies and other modalities is needed. HIAS has provided community-based MHPSS services together with gender-based violence prevention and response programs, economic inclusion, and legal protection for refugees and asylum seekers from Venezuela, Colombia, and other countries in Ecuador since 2003. The initial COVID-19 state of emergency was declared in Ecuador in March 2020, which instituted restrictions on in-person programming and mobility until September 2020 and required HIAS to adjust their community-based MHPSS service delivery model.

To adapt to the evolving COVID-19 situation and policies, HIAS Ecuador developed flexible MHPSS implementation plans to enable remote, in-person, and hybrid program delivery. This included establishing new strategies to increase the reach of HIAS MHPSS programming: a call center that could be accessed across Ecuador for referral to all services; hotlines for psychosocial care and social network platforms for dissemination of information about mental health and COVID-19. Quickly, it became apparent that a significant proportion of refugee, migrant, and host community members were unable to access remote-delivered MHPSS due to lack of connectivity, sharing of mobile phones, or having had to sell their phone to meet basic needs. In response, HIAS engaged in participatory approaches (e.g., individual and group interviews, community planning and consultation meetings held virtually and in-person, depending on local policies, socialization of practices and suggestions for outreach through community leaders) to understand individual needs and develop viable strategies to deliver MHPSS to individuals with the most limited access. Based on their findings, HIAS developed and harnessed a network of *community promoters*- community members who received training in community management, empowerment, COVID-19, and the promotion of healthy behaviors- to disseminate information within their communities in person or through existing remote means of communication, identify and make proactive MHPSS referrals, and continue to advise HIAS on safe in-person delivery spaces and accessible remote delivery strategies. Working with the community enabled HIAS staff to identify individuals they were unable to reach through technology and who required in-person services implemented in safe community spaces or in offices with appropriate COVID-19 precautions.

An example of the delivery strategies that HIAS developed is ‘forum chats’, scheduled group conversations held using WhatsApp. Prior to the scheduled time, HIAS sent a short video explaining the topic of focus and activities to group members to work through asynchronously. Then, group members used the scheduled chat time to synchronously share their experiences via text, voice notes, and photos. This approach required less bandwidth than a group video or audio call, increasing accessibility. In another example HIAS arranged for kits with intervention materials to be delivered to individuals’ homes who could not reach MHPSS services via phone or mobile conferencing/chatting options. With an orientation from a psychologist, the participants were then able to progress through the intervention materials independently, meeting with MHPSS groups and/or the psychologist at least once a month (in person or remotely) to share their progress, challenges they had encountered with the material, and to reinforce the skills and concepts presented in the intervention materials. These flexible strategies enabled HIAS to downscale in-person activities and personalize service delivery strategies to reach populations in need of MHPSS.

The successful implementation of these strategies to increase access to MHPSS services during the COVID-19 state of emergency provide evidence of feasibility. However, future efforts are needed to evaluate the intended and unintended impacts of these strategies on the implementation and effectiveness of MHPSS services in these contexts, including in the post-pandemic phase, and may be guided by implementation science frameworks that incorporate considerations for promoting health equity [45]. 

## Equity in implementation of MHPSS: challenges and strategies for a community-based psychosocial support group for victims of the armed conflict in Colombia

Flexibility in the design of implementation strategies for MHPSS may also require dynamic changes throughout the course of implementation to respond to changes in the context and to maintain participant safety. In late 2020, as part of a larger research study, Heartland Alliance International (HAI) and Universidad de los Andes piloted an adapted community-based psychosocial support group model for conflict survivors in Quibdo, Colombia using in-person, remote, and hybrid modalities. Originally developed in 2010 as part of the Alianza Con Organizaciones Por lo Emocional (ACOPLE) project by HAI, Johns Hopkins University (JHU), the National Association of Displaced Afro-Colombians (AFRODES), and CISALVA Institute of Universidad del Valle [28, 29], this model evolved over time to remain responsive to participant needs, ultimately incorporating enhanced collective problem-solving components and culturally-specific expressive elements such as dance and artwork. The program is facilitated by non-specialist community members (Community Psychosocial Agents, CPAs) who are trained and supervised by mental health professionals. When the pandemic began, raising concerns that in-person services would be unsafe or prevented by lock down, the research team worked with the CPAs to adapt the group protocol to remote and hybrid (four problem-solving sessions held remotely and four expressive sessions held in person) modalities, and in so doing, encountered a range of challenges in ensuring equitable access and safe participation in services.

Like HIAS, the project faced connectivity difficulties, including participants lacking phones and credit, frequent change in phone numbers due to insufficient funds and security concerns (e.g., tracking by armed groups), and spotty or non-existent phone or internet service in rural areas. While students and younger people were generally technology-savvy, older and less educated groups, including both participants and CPAs, were not familiar with smartphones and platforms such as Zoom. Furthermore, applying technology and digital tools specifically created concerns and challenges around maintaining privacy and confidentiality. Despite training, some CPAs tended toward non-confidential use of remote modalities (e.g., personal Facebook and WhatsApp accounts) in an effort to be as accommodating as possible to participants. Most participants joined remote sessions from their homes, allowing for little privacy during discussion of sensitive topics. Calls could have been overheard by family members and neighbors, engendering stigma and creating safety risks. Some families use shared phones, which may be held by male family members, introducing particular risk for participants who live with perpetrators of domestic violence. The use of remote modalities also made it harder to manage certain emergency/high risk situations (e.g., suicidality, domestic violence). Whereas CPAs were trained to assess risk and create related safety plans, this process had historically entailed keeping those who expressed active suicidality or faced immediate threats at home from leaving the session until a safety plan was in place—a critical step that could not be easily maintained during remote service delivery. In addition, many referral agencies, including emergency services, were not fully active, limiting accessibility in critical cases.

In response, the team developed a number of tools and protocols to ensure flexibility, equity, and participant and staff safety. Most importantly, the team determined that participants should be clearly provided with information on available options and associated risks and benefits and then given the choice of whether to participate in person (in small groups with physical distancing precautions) or remotely (with an option to borrow a device from HAI’s smartphone lending library if needed), rather than being offered just one option, which inevitably disadvantaged certain individuals. For example, requiring certain individuals to participate remotely (e.g., rural participants lacking reliable phone service or those who were not technologically-savvy) had potential to prevent or discourage their attendance, while others (e.g., those with health conditions creating vulnerability to COVID-19 or those dependent on crowded public transport) were unlikely to join if only in-person options were available. Prioritizing participant choice and autonomy allowed participants to choose a modality that best suited their particular needs, resources, and comfort level. Given the inherent uncertainty of the situation and the impossibility of fully understanding each individual’s unique combination of risks and needs, it was critical that participants, not service providers, make this decision. CPA facilitators were also able to choose their preferred modality(ies), therefore allowing some CPAs who had to leave the community for security reasons to continue to participate in the project.

Another important adaptation was the development of a tailored safety planning protocol and checklist for remote service delivery [30], including components such as: ensuring CPAs use only work phones and social media accounts and do not save full participant names in phone logs; working with participants to develop the best schedule for private calling; developing code words to assess whether the participant (and not other family members) answer the phone, to signal emergencies, or to signal when someone else is able to overhear; and teaching participants how to erase call histories and save service provider contact info in a non-identifying way. HAI’s existing suicide and self-harm and gender-based violence exposure risk assessments and safety planning protocols were also adapted to the remote context, for instance by instructing service providers to stay on the line with participants while contacting emergency services through a separate line.

A final set of challenges concerned the wellbeing of service providers. HAI’s service providers shared that working remotely was more stressful and less satisfying than working in person. Frequent follow-up with participants to “check in” and encourage group attendance proved exhausting in some cases. A lack of in-person support from colleagues impeded ability to debrief about difficult sessions. To fill these gaps, the team emphasized the importance of ensuring remote access to regular group supervision and to staff support programming for service providers, including through WhatsApp groups and video sessions.

Following a pilot study [31], participants and staff reported feeling generally safe and comfortable in all modalities, and while some lamented connectivity challenges in remote modalities, others shared that these modalities created new opportunities. Some remote participants reported that scheduling flexibility facilitated access, such that participants could join from their workplaces and while staying home with children. Others shared that remote participation was safer in areas with significant community violence, as participants did not have to meet in public places or take public transport. Overall, the team concluded that the addition of a remote option promoted equitable access, but only with extensive training and support and when in-person options were also made available. Additional testing is in process to help determine if these service adaptations are effective in achieving desired participant outcomes, and if they can and should be sustainably implemented as part of regular service delivery post-pandemic.

## Equity in maintenance of MHPSS programming: challenges and adaptations made to ensure continuity in MHPSS services for Rohingya mothers and children in refugee camps in Cox’s Bazar, Bangladesh

Unlike the examples described above in Colombia, Ecuador, and Lebanon, transitioning to remote service delivery may not be possible for some MHPSS interventions and in some contexts, particularly those considered as life saving in humanitarian settings. In-person services, even if more costly or requiring major adaptations, are often favored especially in settings where services are provided only by non-governmental organizations and other resources are scarce. Remote interventions are a last option for consideration. For example, in refugee camps in Cox’s Bazar, Bangladesh, humanitarian donors have supported both in-situ and remote adaptations, with both presenting some logistic and administrative challenges and constraints for service users and personnel.

Since 2017, following the influx of Rohingya refugees in Bangladesh, Action contre la Faim (ACF) has implemented Baby Friendly Space (BFS) programs [32] in refugee camps, which provide comprehensive MHPSS services to lactating and pregnant women and their infants under two to improve maternal mental health and childcare practices, as well as to malnourished children under 5 years old. Children are referred from Integrated Nutrition Centers where they receive nutrition treatment to BFS services. BFS services include provision of information on topics such as breastfeeding, child stimulation, and psychological support in individual and group sessions. Drawing on ACF’s expertise adapting to emergency contexts, it was quickly decided to balance remote counseling services with an in-person field response following the onset of the COVID-19 pandemic to minimize infection as much as possible while still ensuring basic needs were met. As nutrition and health services were considered essential for Rohingya refugees, under the rules imposed by the Ministry of Health and Family Welfare of Bangladesh, BFS services remained operational, but adaptations had to be made to ensure safe in-person services for the mothers and children who were the most in need of MHPSS. Whenever possible, ACF aimed at maintaining partial in-person presence of psychosocial workers and community volunteers who were already trained in Psychological First Aid [33], case management, and psychosocial support activities. 

After this first adaptation phase, ACF began to resume operation under pre-pandemic work modalities in order to restore fuller access to BFS. New standard operating procedures, such as for Personal Protective Equipment, were given to staff and service users regarding their health and protection. ACF facility-based workers complied with arrangements and protocols recommended by the Government of Bangladesh and WHO in the refugee camps at all times for the project staff and beneficiaries accessing services, which led to an increase amount of time spent during and between consultations. As a consequence, a smaller number of participants attended group activities, individual counseling was promoted, and referral pathways between the different activities were reorganized.

## Discussion

The adaptations discussed above have important actionable implications for equity. In some cases such as the UNICEF and WHO COVID-19 MHPSS online consultations, transitions to remote delivery platforms and heightened focus on mental health have increased the availability of information, accessibility of MHPSS services, and opportunities to participate in service design. However, as was observed in Ecuador, these changes may exacerbate existing inequities in access to care, access to knowledge, and inclusion in co-design and consultation processes that can impact the care received. These opposing outcomes highlight the importance of engaging communities when designing programs and implementation strategies to understand potential unintended consequences while also systematically monitoring the impact of these adaptations on health equity.

While great efforts have been made to adapt mental health promotion, prevention, and treatment programs through innovative methods, there are often barriers to the accessibility of such approaches for the most marginalized populations (e.g., migrants in transit, women in contexts in which phones are shared or held by male family members, youth, elderly people, people living with disabilities, those from minority language groups, or those without access to internet, network coverage, devices, or technology skills) [34, 35]. Providing resources (e.g. phone lending libraries, credit/internet top-ups) and training in technological literacy and reducing reliance on literacy for remote modalities can enhance accessibility of remote options. Safeguards to ensure safety and confidentiality during use of remote modalities must be considered in context with input from communities and prioritized in the design or redesign of services.

Perhaps most importantly, rather than a one-size fits all approach to adapting MHPSS programs during the pandemic and beyond, locally developed and contextualized solutions, including flexible, multi-option solutions in which participants are empowered to choose based on their situation and needs are necessary to ensure relevant and effective programs are delivered equitably. When possible, programming should incorporate innovations that participants already utilize and are comfortable with (e.g., Whatsapp voice notes). To ensure accountability to participants and equitable access, some programs may decide to implement remote solutions while also maintaining in-person or hybrid services (with safeguards). In these cases, programs should educate participants about options, risks, and benefits and then let them decide which modalities work best for them, as suggested in the Colombia case study. Pro-equity investments that help overcome the digital divide are needed given the increased reliance on remote delivery of MHPSS.

As our understanding of the pandemic, the response, and its effect grow and change, researchers and practitioners must measure and document adaptations and the impact that adaptations to MHPSS implementation have on equity and MHPSS outcomes, expected and unexpected. Many of these strategies may also be applied to address inequities in participation, access, and implementation of MHPSS outside of the COVID-19 pandemic context. For instance, some strategies, such as the strictly remote service delivery and frequent remote follow-up with participants described in Colombia, that were designed to promote increased access to services for vulnerable populations can simultaneously perpetuate fatigue or burnout amongst providers. On the contrary, as experienced in Rohingya refugee camps in Bangladesh, some strategies may be employed to increase feasibility of maintaining services during periods of challenging circumstances (e.g., restrictions imposed on in-person services during peaks of the COVID-19 pandemic). They may simultaneously impose barriers to vulnerable populations, such as those lacking access to and/or comfort with technology or create opportunities to empower them. Meanwhile, some adaptations, such as use of diverse, participant-preferred communication tools (e.g., Whatsapp voice notes) and allowing participants to choose their intervention modality (e.g., remote, in-person, or hybrid), made specifically to accommodate pandemic-related restrictions may unexpectedly prove beneficial to equitable access even in post-pandemic contexts. Implementation strategies should account for potentially differential impacts on all actors, including program participants, implementation staff, policymakers, and funders.

This manuscript has outlined several adaptations that have been implemented, but to advance equity we need to better understand how effective, efficient, feasible, and acceptable adaptations are in short-term and long-term. Future research should seek to explore this. An implementation science approach can help generate an understanding of conflicting priorities of different stakeholders (for individual, within communities, among stakeholders) and from an equity perspective, can help sensitize stakeholders to the range of priorities amongst different stakeholders. Processes that examine stakeholder priorities and the implications of addressing them or not are needed to ensure equitable access to services and outcomes. Within the processes, an emphasis towards implementation strategies that aim to overcome barriers and address priorities of those who are underserved can help ensure that populations are not further marginalized by compounded effects of the pandemic and a potentially inequitable response. Integrating both co-creation and participatory monitoring and evaluation processes are key to ensuring ongoing input and feedback about adaptations and their outcomes from all stakeholders—participants, service providers, community leaders, and other actors.

Systematic documentation of the implementation process, including adaptations and their outcomes, not only provides data to inform active learning and adaptation for an immediate response, but, when disseminated, also facilitates more rapid learning in future scenarios that require adaptation and informs future “routine” implementation and possible scale-up and scale-out. Documentation and dissemination of inclusion considerations that lead to adaptations or selection of implementation processes, and the impacts of those resulting processes, may more effectively inform and ensure inclusive development and implementation of services, programs, and policies.

The COVID-19 pandemic has highlighted the importance of preventing and treating MHPSS-related issues and has created opportunities for innovative programming approaches and new research that addresses commonly overlooked problems and has the potential to improve the quality of services provided even beyond the COVID-19 emergency context [36–39]. Access to knowledge and the necessity to provide MHPSS services has heightened alongside growing global interest from communities of practice within health and other sectors. The case studies presented in this paper provide examples of such opportunities. Donors’ awareness of the importance of funding MHPSS programming, which is increasingly supported by Ministries of Health worldwide [40–42], has also grown. In this time of increased needs, awareness, and opportunities, it is essential that we consider equity issues in all stages of program design, implementation, and evaluation of MHPSS services. Existing equity frameworks [43–45] could serve as tools to operationalize equity considerations, as we consider adopting innovations into routine practice in order for MHPSS to truly build back better and fairer. 

## Conclusion

The COVID-19 pandemic has had devastating consequences on economies, health, and wellbeing globally. The negative impacts have not been equitably distributed, with evidence that existing disparities have been exacerbated, disproportionately impacting the most vulnerable, including those living in humanitarian contexts. One positive consequence of the pandemic is that public, policy, and donor attention towards mental health and the importance of MHPSS services has increased significantly. It is vital that we use the momentum and attention generated around MHPSS services during the COVID-19 pandemic to continue to build and improve existing services for vulnerable populations. Increased awareness will be limited in its impact if it is not accompanied by increased accessibility and availability, quality assurance, and fostering of equity. We should use this moment to expand MHPSS services and better integrate MHPSS into other health and social services to reach more people, but in so doing must engage vulnerable and marginalized affected communities in decision making, planning, and implementation to ensure their access to participation and benefits and remain accountable to their needs. As a field, a critical first step to the process of “Building Back Fairer” is to document and share lessons learned on the rapid adaptations required for MHPSS services in order to identify best practices for rapid adaptation of implementation strategies while ensuring equity and accountability, particularly strategies that can be maintained to continue to increase equity as the pandemic changes and recedes. We should also actively seek to move beyond documenting and sharing to actual learning and adapting our process and systems for longer-term gains. The creativity and adaptability that have been a hallmark of successful humanitarian action, have been further demonstrated in the recent years. This creativity and intersectoral learning can serve as a launching point to build greater resiliency within programs, organizations, and, most importantly, communities.

## Data Availability

Not applicable.

## Abbreviations

ACF: Action contre la Faim
ACOPLE: Alianza Con Organizaciones Por lo Emocional
AFRODES: National Association of Displaced Afro-Colombians
BFS: Baby Friendly Spaces
CAB: Community advisory board
CPA: Community Psychosocial Agents
GIZ: Deutsche Gesellschaft für Internationale Zusammenarbeit
HAI: Heartland Alliance International
HEARD: Health Evaluation and Applied Research Development
IS: Implementation science
JHU: Johns Hopkins University
MHPSS: Mental health and psychosocial support
PFA: Psychological First Aid
PM+: Problem Management Plus
UNICEF: United Nations Children’s Fund
USAID: United States Agency for International Development
WHO: World Health Organization
